# Dipeptidyl Peptidase-4 Inhibitor Increases Vascular Leakage in Retina through VE-cadherin Phosphorylation

**DOI:** 10.1038/srep29393

**Published:** 2016-07-06

**Authors:** Choon-Soo Lee, Yun Gi Kim, Hyun-Jai Cho, Jonghanne Park, Heewon Jeong, Sang-Eun Lee, Seung-Pyo Lee, Hyun-Jae Kang, Hyo-Soo Kim

**Affiliations:** 1 National Research Laboratory for Stem Cell Niche, Seoul National University College of Medicine, 101 Daehak-ro, Jongno-gu, Seoul, Korea; 2 Innovative Research Institute for Cell Therapy, Seoul National University Hospital, 101 Daehak-ro, Jongno-gu, Seoul, Korea; 3 Department of Molecular Medicine and Biopharmaceutical Sciences, Graduate School of Convergence Science and Technology, and College of Medicine or College of Pharmacy, Seoul National University, 101 Daehak-ro, Jongno-gu, Seoul, Korea; 4 Cardiovascular Center & Department of Internal Medicine, Seoul National University Hospital, 101 Daehak-ro, Jongno-gu, Seoul, Korea.

## Abstract

The inhibitors of CD26 (dipeptidyl peptidase-4; DPP4) have been widely prescribed to control glucose level in diabetic patients. DPP4-inhibitors, however, accumulate stromal cell-derived factor-1α (SDF-1α), a well-known inducer of vascular leakage and angiogenesis both of which are fundamental pathophysiology of diabetic retinopathy. The aim of this study was to investigate the effects of DPP4-inhibitors on vascular permeability and diabetic retinopathy. DPP4-inhibitor (diprotin A or sitagliptin) increased the phosphorylation of Src and vascular endothelial-cadherin (VE-cadherin) in human endothelial cells and disrupted endothelial cell-to-cell junctions, which were attenuated by CXCR4 (receptor of SDF-1α)-blocker or Src-inhibitor. Disruption of endothelial cell-to-cell junctions in the immuno-fluorescence images correlated with the actual leakage of the endothelial monolayer in the transwell endothelial permeability assay. In the Miles assay, vascular leakage was observed in the ears into which SDF-1α was injected, and this effect was aggravated by DPP4-inhibitor. In the model of retinopathy of prematurity, DPP4-inhibitor increased not only retinal vascularity but also leakage. Additionally, in the murine diabetic retinopathy model, DPP4-inhibitor increased the phosphorylation of Src and VE-cadherin and aggravated vascular leakage in the retinas. Collectively, DPP4-inhibitor induced vascular leakage by augmenting the SDF-1α/CXCR4/Src/VE-cadherin signaling pathway. These data highlight safety issues associated with the use of DPP4-inhibitors.

Diabetic retinopathy is a major cause of blindness among working age adults^1^,^2^. It is classified into two stages: non-proliferative and proliferative. The pathophysiology of non-proliferative diabetic retinopathy involves increased retinal vascular permeability, alterations in retinal blood flow, and abnormal retinal microvasculature, all of which lead to retinal ischemia. The appearance of neovascularization in response to retinal hypoxemia is the hallmark of proliferative diabetic retinopathy.

Stromal cell derived factor-1α (SDF-1α) is a member of the CXC chemokine subfamily^3^. SDF-1α, through its receptor CXCR4, activates Src^3^,^4^,^5^,^6^,^7^, which in turn induces the phosphorylation and disruption of vascular endothelial-cadherin (VE-cadherin)^8^,^9^,^10^, a critical process regulating angiogenesis and vascular permeability^11^,^12^,^13^,^14^. SDF-1α is increased in damaged tissue and promotes tissue repair and angiogenesis^4^,^15^. Thus, promising results have been achieved through the direct injection of SDF-1α or via gene delivery approaches^15^,^16^,^17^. However, SDF-1α might aggravate diabetic retinopathy because angiogenesis and increased vascular permeability are the key pathophysiologies of diabetic retinopathy. Butler *et al.* have demonstrated that SDF-1α induces retinopathy in a murine model and that injection of antibodies to SDF-1α prevents retinal neovascularization^2^. Previous reports have indicated that SDF-1α also increases vascular permeability^18^,^19^.

CD26 (dipeptidyl peptidase-4; DPP4) is an antigenic enzyme expressed on the surface of most cell types, and it is also found as a catalytically active soluble form in plasma^20^,^21^. DPP4 cleaves N-terminal dipeptides, i.e., proline or alanine residues, from peptides. Importantly, DPP4 inactivates SDF-1α by cleaving specific amino acids^22^,^23^. This process renders SDF-1α biologically inactive but still able to bind to CXCR4 and block active SDF-1α from binding to CXCR4^24^,^25^. Consequently, the inhibition of DPP4 stabilizes biologically active SDF-1α, as demonstrated in both animal models^20^,^26^,^27^ and human diabetic patients^28^,^29^. DPP4-inhibitors are a new class of oral hypoglycemics. The prescription of these drugs to treat diabetes mellitus has increased enormously over the past several years. However, DPP4-inhibitors might have adverse effects on diabetic retinopathy by promoting vascular leakage because DPP4-inhibitors increase active SDF-1α concentration which would activate the SDF-1α/CXCR4/Src pathway. Activation of the SDF-1α/CXCR4/Src pathway might induce disruption of the VE-cadherin-catenin complex, a critical component in maintaining endothelial cell-to-cell junction integrity^30^,^31^. We tested the hypothesis of whether DPP4-inhibitors, by stimulating the SDF-1α/CXCR4 axis and subsequently resulting in Src-mediated phosphorylation of VE-cadherin, would increase vascular permeability, which is a key process in diabetic retinopathy.

## Results

### The effects of H/R (hypoxia/reoxygenation) on the expression levels of SDF-1α, CXCR4, and DPP4 in human vascular cells: endothelial and vascular smooth muscle cells

**Figure 1:**
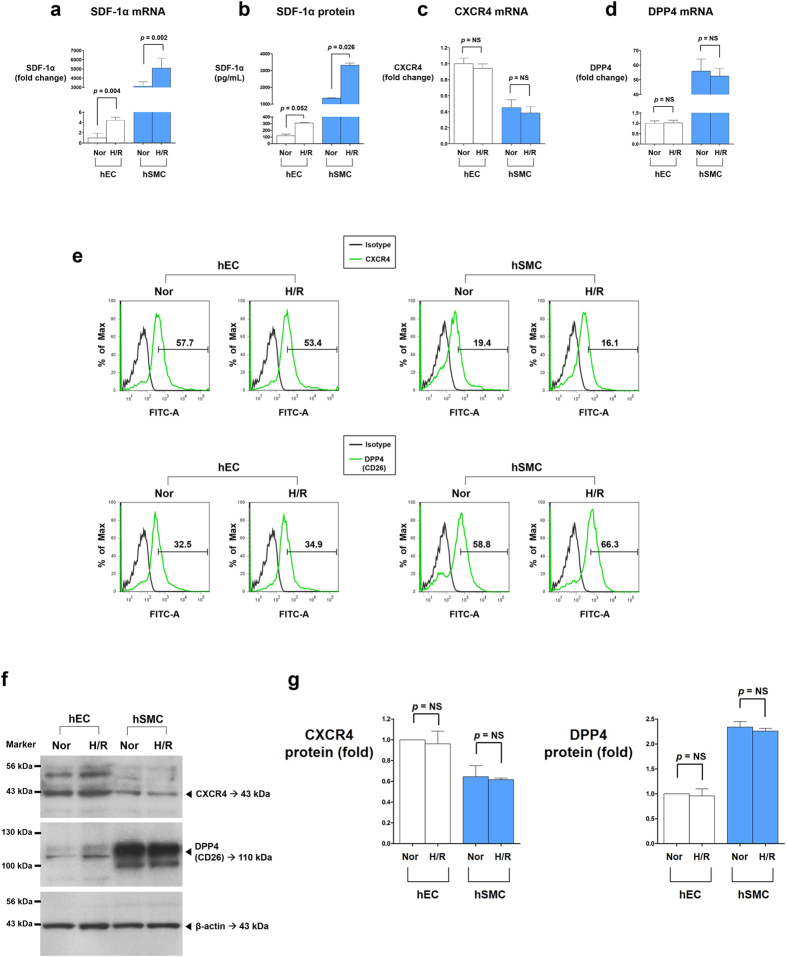
Expression of SDF-1α, CXCR4, and DPP4 in human vascular cells under normoxia and H/R. The main source of SDF-1α was hSMCs, while its receptor CXCR4 was mainly expressed on hECs. DPP4, a regulatory enzyme to degrade SDF-1α, was mainly expressed in hSMCs. H/R significantly increased SDF-1α expression compared with normoxic condition in both hECs and hSMCs. However, H/R did not affect the mRNA or protein expression levels of CXCR4 and DPP4 in both hECs and hSMCs. **(a)** RT-qPCR analysis of SDF-1α mRNA (n = 6). Bars represent relative quantification values. Error bars denote relative quantification maximum values. **(b)** ELISA of SDF-1α protein in culture supernatants (n = 2). Data are shown as means ± SD. **(c)** RT-qPCR analysis of CXCR4 mRNA (n = 6). Bars represent relative quantification values. Error bars denote relative quantification maximum values. **(d)** RT-qPCR analysis of DPP4 mRNA (n = 6). Bars represent relative quantification values. Error bars denote relative quantification maximum values. **(e)** FACS analysis of CXCR4 and DPP4 protein on the surface of hECs and hSMCs. **(f)** Western blot analysis of CXCR4 and DPP4. **(g)** Quantification graphs of the western blot (n = 4). Data are shown as means ± SD. *p* values are determined by Student’s *t* test. DPP4: dipeptidyl peptidase-4; hECs: human endothelial cells; hSMCs: human vascular smooth muscle cells; H/R: hypoxia/reoxygenation; Nor: normoxia; SD: standard deviations; SDF-1α: stromal cell derived factor-1α.

We examined the expression levels of SDF-1α, its receptor CXCR4, and DPP4 in human vascular cells. Under normoxic conditions, SDF-1α expression was higher in hSMCs (human smooth muscle cells) than it was in hECs (human endothelial cells) at the mRNA and secreted protein levels (Fig. 1a,b), whereas the expression of its receptor CXCR4 was higher in hECs than in hSMCs at the mRNA and cell surface protein levels (Fig. 1c,e). DPP4, which degrades SDF-1α, was higher in hSMCs than in hECs at the mRNA and cell surface protein levels (Fig. 1d,e).

When these cells were exposed to H/R stress, SDF-1α was significantly induced, suggesting that SDF-1α is a hypoxia-responsive molecule (Fig. 1a,b). In contrast, mRNA (Fig. 1c,d) and protein levels (Fig. 1e–g) of CXCR4 and DPP4 did not change in response to H/R in either hECs or hSMCs. These results suggested the presence of both paracrine and autocrine networks in which SDF-1α, mainly secreted from hSMCs, stimulates CXCR4 mainly on endothelial cells, an effect augmented by H/R. DPP4-inhibitors might also play a role in modulating the SDF-1α/CXCR4 system in vascular cells because both cell types stably express DPP4.

### Disruption of endothelial integrity by H/R

**Figure 2:**
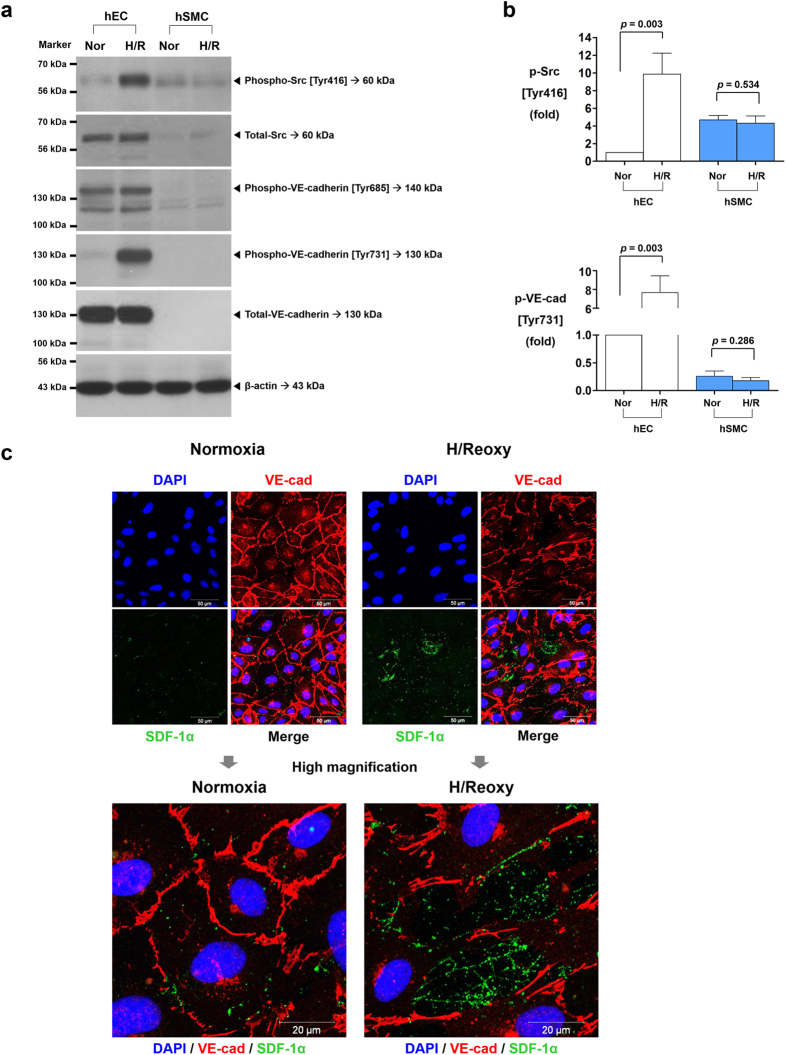
Activation of Src kinase during H/R leads to VE-cadherin [Tyr731] phosphorylation and disruption of cell-to-cell junctions. H/R activated Src kinase mediated phosphorylation of VE-cadherin [Tyr731], leading to disruption of cell-to-cell junctions in hECs monolayer culture, suggesting the existence of autocrine pathway of the SDF-1α/CXCR4/Src/VE-cadherin signaling cascade in hECs. **(a)** Western blot analysis of phosphorylated Src and VE-cadherin under normoxia and H/R. **(b)** Quantification graphs of the western blot (n = 3). **(c)** Nuclei (blue), VE-cadherin (red), and SDF-1α (green) were separately stained and merged. H/R disrupted VE-cadherin cell-to-cell junctions, which coincided with the SDF-1α expression. All data are shown as means ± SD. *p* values are determined by Student’s *t* test. DAPI: 4′,6-diamidino-2-phenylindole; H/Reoxy: hypoxia/reoxygenation; p-Src: phosphorylated Src; p-VE-cad: phosphorylated VE-cadherin; Tyr: tyrosine; VE-cadherin and VE-cad: vascular endothelial-cadherin. Other abbreviations as in Fig. 1.

Interaction of SDF-1α with its receptor CXCR4 activates Src^3^,^4^,^5^,^6^,^7^, which in turn induces the phosphorylation and disruption of VE-cadherin. Therefore, we examined the influence of H/R on this signaling pathway. According to our data, the main source of SDF-1α was hSMCs, whereas CXCR4 was mainly expressed in hECs. Therefore, we tested the paracrine network between the two cell types by using an *in-vitro* model with transwell, in which two types of cells were co-cultured without direct contact: hSMCs were placed in the upper chamber, and hECs were placed in the lower chamber. H/R on hSMCs significantly increased the phosphorylation of Src [Tyr416] and VE-cadherin [Tyr731] in hECs, but this effect was prevented by either CXCR4-blocker (AMD3100) or Src-inhibitor (PP2) (Supplementary Fig. S1). In contrast with [Tyr731] of VE-cadherin, phosphorylation at [Tyr685] was not affected by the SDF-1a/CXCR4/Src pathway. These results suggested that SDF-1α secreted from hSMCs under H/R stimulated its receptor CXCR4 on hECs and then induced phosphorylation of Src [Tyr416] and VE-cadherin [Tyr731] in hECs. SDF-1α was also secreted from hECs (Fig. 1a,b). Thus, we checked the autocrine pathway by using hECs monolayer culture. H/R, a strong inducer of SDF-1α also in hECs, significantly increased Src [Tyr416] phosphorylation and subsequently VE-cadherin [Tyr731] phosphorylation in hECs monolayer culture (Fig. 2a,b). In consistency with the previous data, phosphorylation of VE-cadherin [Tyr685] was not changed in response to H/R (Fig. 2a). In hSMCs mono-culture experiment, the phosphorylation of Src [Tyr416] and VE-cadherin [Tyr685, Tyr731] was not induced by H/R (Fig. 2a,b).

In the immuno-fluorescence staining of hECs, H/R increased the immuno-staining of SDF-1α and induced the disruption of endothelial cell-to-cell junctions labeled with VE-cadherin (Fig. 2c; quantification of the fluorescence intensity of VE-cadherin and SDF-1α is shown in Supplementary Fig. S2). Interestingly, the disruption of endothelial cell-to-cell junctions mainly occurred at the sites where SDF-1α immuno-reactivity was strong in the merged image of the two separate immuno-fluorescence stainings of SDF-1α and VE-cadherin.

### Disruption of integrity and increased permeability of endothelial cells by DPP4-inhibitor

**Figure 3:**
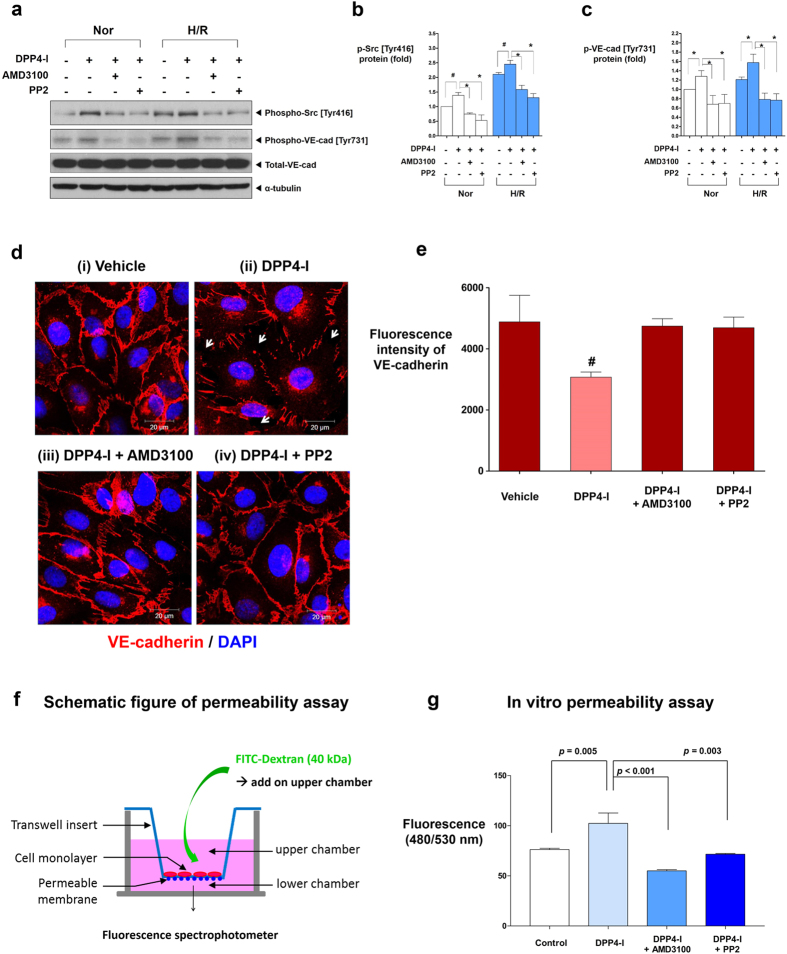
DPP4-inhibitor induces phosphorylation of Src [Tyr416] and VE-cadherin [Tyr731] which leads to cell-to-cell junction disruption and endothelial leakage. **(a)** DPP4-inhibitor (DipA; 100 μM) induced the phosphorylation of Src [Tyr 416] and VE-cadherin [Tyr731] in hECs in both normoxia and H/R conditions, which was reversed by CXCR4-blocker (AMD3100; 1 μg/ml) or Src-inhibitor (PP2; 1 μM). **(b,c)** Quantification graphs of the western blot (*p < 0.01, ^#^*p* < 0.05; n = 3–5). **(d)** Confluent cultures of hECs were either (i) left untreated, (ii) treated with DPP4-inhibitor, (iii) pre-treated with AMD3100 and then treated with DPP4-inhibitor, (iv) pre-treated with PP2 and then treated with DPP4-inhibitor. VE-cadherin cell-to-cell junctions were disrupted after treatment with DPP4-inhibitor, which was prevented by CXCR4-blocker or Src-inhibitor. **(e)** Fluorescence intensity quantification of VE-cadherin of (**d**) (^#^*p* < 0.05 compared to all other groups; n = 3 for each group). **(f)** Experimental scheme of endothelial permeability assay. **(g)** Endothelial permeability is increased after adding DPP4-inhibitor to the upper chamber which was attenuated by CXCR4-blocker or Src-inhibitor pre-treatment (n = 5 for each group; molecular weight of FITC-dextran: 40 kDa; DipA treatment time: 60 min; time for permeance: 20 min). All data are shown as means ± SD. *p* values are determined by Student’s *t* test. DipA: diprotin A; DPP4-I: dipeptidyl peptidase-4 inhibitor; FITC-dextran: fluorescein isothiocyanate conjugated-dextran. Other abbreviations as in Figs 1 and 2. White arrows indicate cell-to-cell junction breakage.

To test whether DPP4-inhibitors would increase vascular permeability, we treated hECs with diprotin A (DipA; a chemical inhibitor of DPP4) and assessed the phosphorylation of Src and VE-cadherin. H/R on hECs induced the phosphorylation of Src [Tyr 416] and VE-cadherin [Tyr731] (Fig. 3a–c). The phosphorylation of Src [Tyr416] and VE-cadherin [Tyr731] was further increased by DPP4-inhibitor. To investigate whether the phosphorylation of VE-cadherin [Tyr731] by DPP4-inhibitor is mediated by SDF-1α/CXCR4 ligand/receptor interaction and the downstream signaling molecule Src kinase, we assessed the inhibitory effects of CXCR4-blocker (AMD3100) and Src-inhibitor (PP2) on the phosphorylation of Src and VE-cadherin. Both CXCR4-blocker and Src-inhibitor reduced the DPP4-inhibitor-induced phosphorylation of Src [Tyr416] and VE-cadherin [Tyr731] in hECs (Fig. 3a–c). We then performed immuno-fluorescence staining for VE-cadherin in hECs to determine whether the DPP4-inhibitor-induced phosphorylation of VE-cadherin actually led to a disruption of endothelial integrity. DPP4-inhibitor disrupted endothelial cell-to-cell junctions labeled with VE-cadherin. This disruption of junctions was prevented by CXCR4-blocker or Src-inhibitor (Fig. 3d,e). Step by step, we evaluated the correlation of disrupted endothelial cell-to-cell junctions on immuno-fluorescence images and the ‘actual leakage’ of the endothelial monolayer, using an *in-vitro* transwell endothelial permeability assay. Endothelial permeability was determined by measuring FITC-dextran (fluorescein isothiocyanate conjugated-dextran; 40 kDa) passage through the endothelial monolayer from the upper to lower chamber (Fig. 3f; see Supplementary Fig. S3 for detailed experimental scheme). The FITC-dextran content in the lower chamber, which represents endothelial permeability, was significantly increased after adding DPP4-inhibitor to the upper chamber, which was prevented by CXCR4-blocker or Src-inhibitor treatment (Fig. 3g).

**Figure 4:**
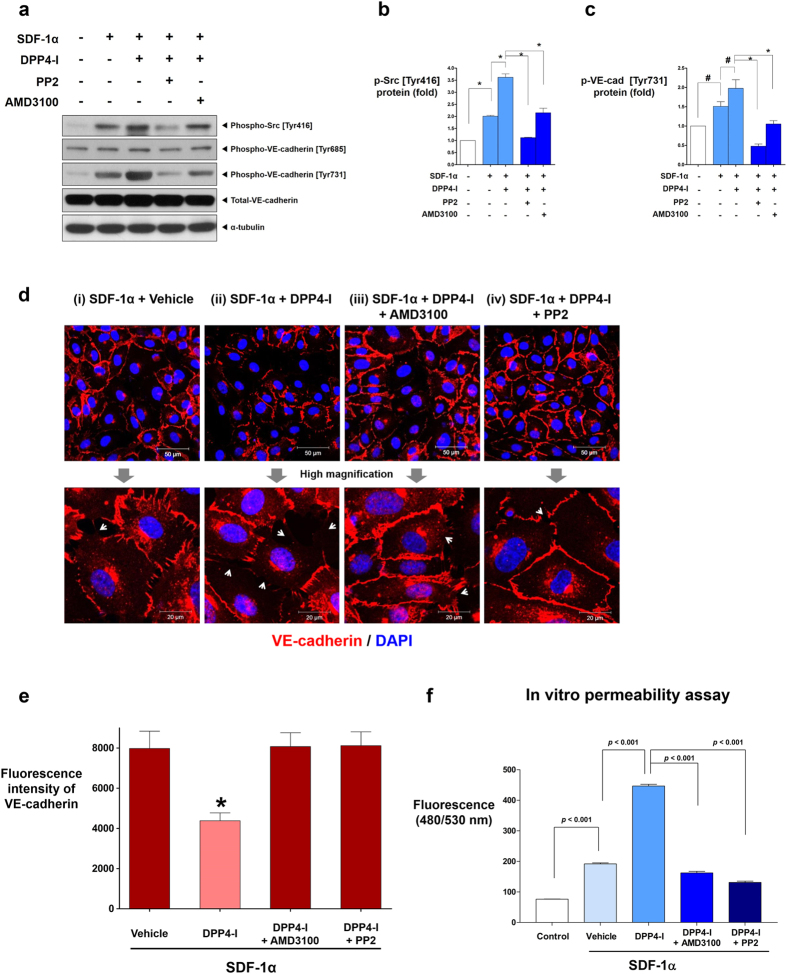
The effects of DPP4-inhibitor under the influence of SDF-1α. **(a)** SDF-1α (100 μM) increased the phosphorylation of Src [Tyr416] and VE-cadherin [Tyr731] which was further augmented by DPP4-inhibitor (DipA; 100 μM). CXCR4-blocker (AMD3100; 1 μg/ml) and Src-inhibitor (PP2; 1 μM) blocked the effects of SDF-1α and DPP4-inhibitor. **(b,c)** Quantification graphs of the western blot (*p < 0.01, ^#^*p* < 0.05; n = 3 for each group). **(d)** The effects of DPP4-inhibitor on VE-cadherin cell-to-cell junction integrity under the influence of SDF-1α is shown. VE-cadherin cell-to-cell junctions were further disrupted by adding DPP4-inhibitor after SDF-1α treatment, which was attenuated by CXCR4-blocker or Src-inhibitor pre-treatment. **(e)** Fluorescence intensity quantification of VE-cadherin of (**d**) (*p < 0.01 compared to all other groups; n = 3 for each group). **(f)** The results of the *in-vitro* transwell endothelial permeability assay. SDF-1α alone increased endothelial permeability. Combination of DPP4-inhibitor with SDF-1α further increased endothelial permeability. CXCR4-blocker and Src-inhibitor pre-treatment attenuated the effects of SDF-1α and DPP4-inhibitor on endothelial permeability (n = 5 for each group; molecular weight of FITC-dextran: 40 kDa; DipA treatment time: 60 min; time for permeance: 20 min). All data are shown as means ± SD. *p* values are determined by Student’s *t* test. Abbreviations as in Figs 1, 2, 3. White arrows indicate cell-to-cell junction breakage.

SDF-1α treatment alone also induced the phosphorylation of Src [Tyr 416] and VE-cadherin [Tyr731] but not the phosphorylation of VE-cadherin [Tyr685] (Fig. 4a–c). The combination of SDF-1α and DPP4-inhibitor was the strongest inducer of Src [Tyr 416] and VE-cadherin [Tyr731] phosphorylation, and this effect was prevented by CXCR4-blocker or Src-inhibitor (Fig. 4a–c). Immuno-fluorescence staining indicated that SDF-1α alone disrupted VE-cadherin cell-to-cell junctions, and this effect was aggravated by DPP4-inhibitor but was prevented by CXCR4-blocker or Src-inhibitor treatment (Fig. 4d,e). *In-vitro* transwell endothelial permeability assay demonstrated that SDF-1α significantly increased endothelial leakage by more than 2-fold. Treatment with DPP4-inhibitor along with SDF-1α treatment further increased endothelial leakage by another 2.5-fold. However, the leakage was prevented by CXCR4-blocker or Src-inhibitor treatment (Fig. 4f). Collectively, these results suggested that the SDF-1α/CXCR4/Src signaling pathway is the mechanism by which DPP4-inhibitor induces the phosphorylation of VE-cadherin [Tyr731] and disrupts endothelial cell-to-cell junctions, leading to actual leakage of the endothelial monolayer.

### Endothelial permeability in multiple doses of DipA and sitagliptin

**Figure 5:**
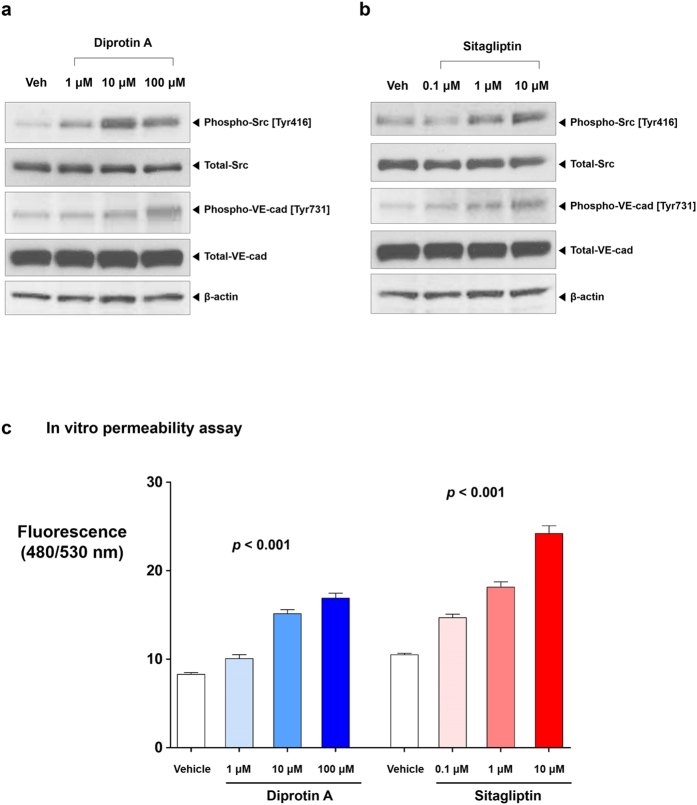
Dose-response relationships of DipA and sitagliptin. DipA **(a)** and sitagliptin **(b)** increased the phosphorylation of Src [Tyr416] and VE-cadherin [Tyr731] in a dose dependent manner. **(c)** DipA and sitagliptin increased endothelial permeability in a dose dependent manner. *p* values of 6 possible pair-wise comparisons were all <0.01 for both groups (n = 5 for each group; molecular weight of FITC-dextran: 40 kDa; DipA and sitagliptin treatment time: 30 min; time for permeance: 5 min). All data are shown as means ± SD. Analysis of covariance with post hoc analysis using Bonferroni’s correction was applied for statistical analysis. Veh: vehicle. Other abbreviations as in Figs 1, 2, 3.

To explore the dose-response relationship, we tested multiple doses of DipA (1, 10, 100 μM). Since DPP4 inhibition is achieved through gliptins in diabetic patients, we also investigated the effects of sitagliptin on the phosphorylation of Src and VE-cadherin and endothelial permeability. Sitagliptin concentrations of 0.1, 1, and 10 μM was used to reflect the actual plasma concentrations in human volunteers taking sitagliptin 25, 100, and 600 mg, respectively^32^, DipA and sitagliptin increased the phosphorylation of Src [Tyr 416] and VE-cadherin [Tyr731] in a dose-dependent manner (Fig. 5a,b). Endothelial permeability was also increased in a dose-dependent manner (*p* < 0.001 for both DipA and sitagliptin; Fig. 5c). The *p* values of 6 possible pair-wise comparisons were all <0.01 for both groups.

### *In-vivo* endothelial leakage by DPP4-inhibitor

**Figure 6:**
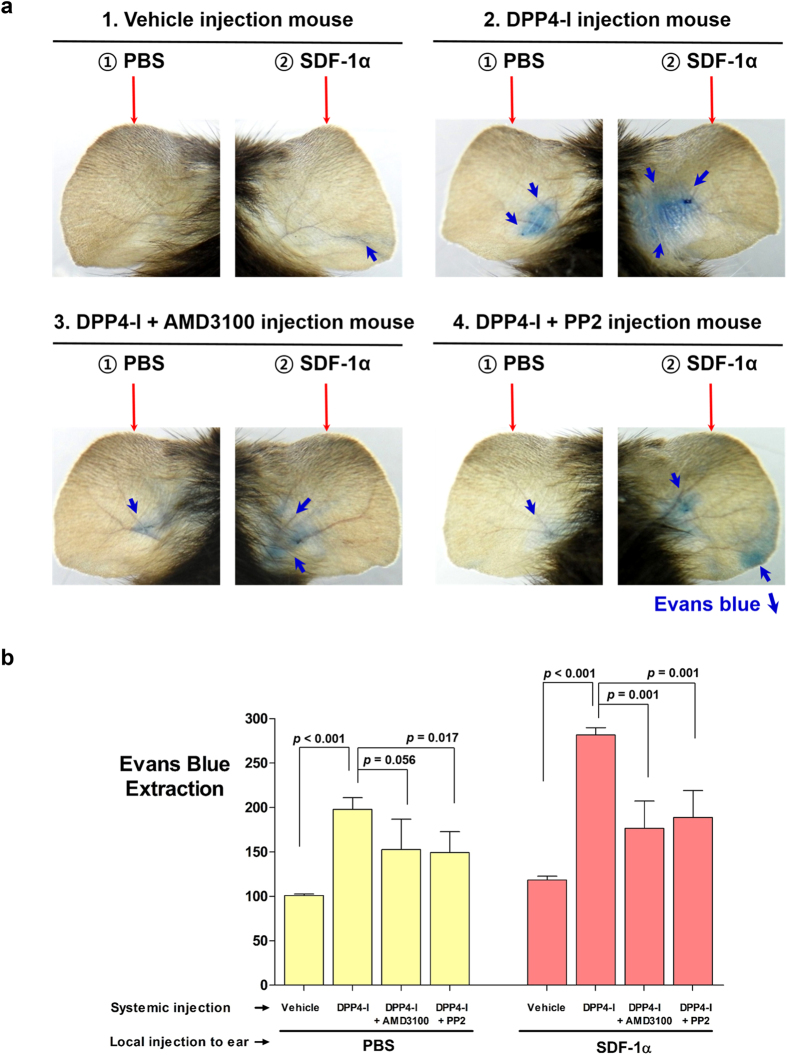
The Miles assay - *in-vivo* effects of DPP4-inhibitor. **(a)** The photographs of both ears of mice under various conditions. Leakage of the dye to the adjacent tissue was seen after treatment with SDF-1α and DPP4-inhibitor, which was reduced by CXCR4-blocker or Src-inhibitor. **(b)** Evans blue dye extravasation to the adjacent tissue was quantified (n = 3–5 for each group). All data are shown as means ± SD. *p* values are determined by Student’s *t* test. Abbreviations as in Figs 1, 2, 3.

In order to evaluate whether DPP4-inhibitors increase vascular permeability *in-vivo*, we performed the Miles permeability assay, using the ears of mice (Supplementary Fig. S4). The ears into which SDF-1α was injected turned blue owing to the extravasation of Evans blue dye, which was systemically administered (Fig. 6a,b). The leakage induced by SDF-1α was aggravated by the intra-peritoneal administration of DPP4-inhibitor (DipA) for 5 days. However, vascular leakage by DPP4-inhibitor and SDF-1α was significantly diminished by CXCR4-blocker (AMD3100) or Src-inhibitor (PP2) (Fig. 6a,b).

### Retinal capillary leakage due to DPP4-inhibitor: retinopathy of prematurity model

**Figure 7:**
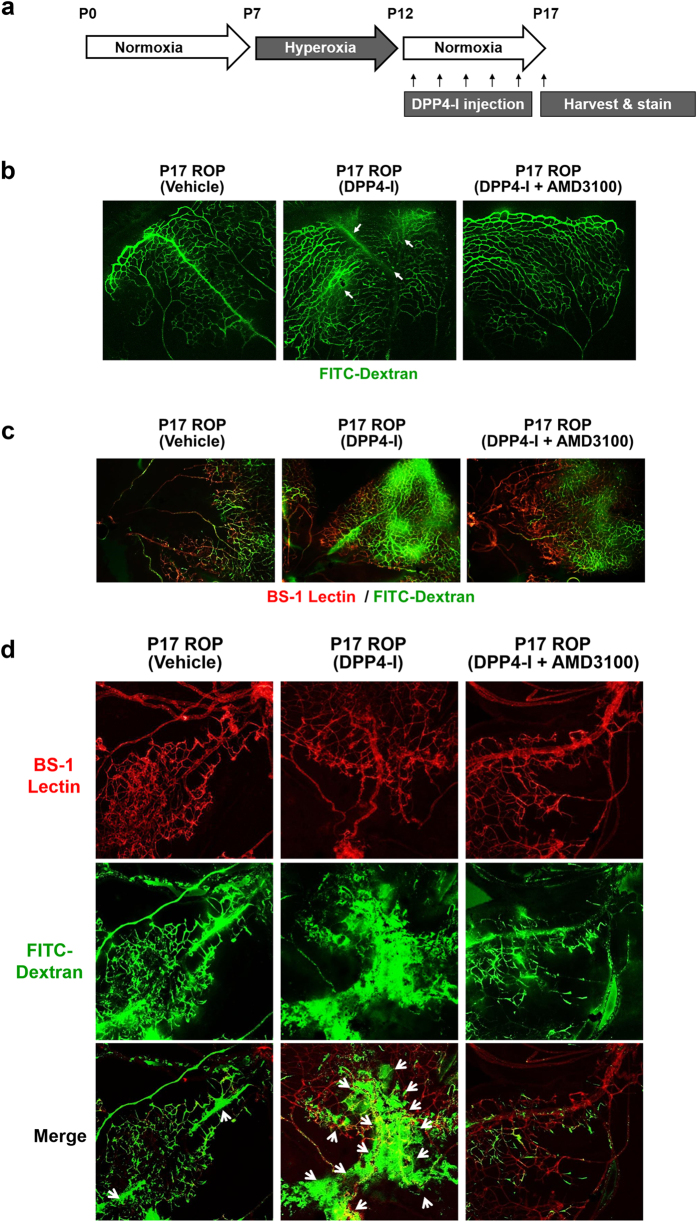
The effects of DPP4-inhibitor on retinal vasculature - retinopathy of prematurity model. **(a)** Schematic illustration of the experiment. **(b)** Compared with the vehicle, DPP4-inhibitor (DipA; 70 μg/kg twice daily) made aberrant and leaky vessels in P17 retina, which was prevented by CXCR4-blocker (AMD3100; 7.5 mg/kg). **(c,d)** All micrographs are merged confocal images of the retinal flat mounts. Evident extravasation of FITC-dextran was demonstrated in the DPP4-inhibitor treated P17 retina compared to the P17 vehicle. CXCR4-blocker treatment neutralized the effects of DPP4-inhibitor. BS-1 lectin: *Bandeiraea simplicifolia* lectin 1; P0: postnatal day 0; P7: postnatal day 7; P12: postnatal day 12; P17: postnatal day 17; ROP: retinopathy of prematurity. Other abbreviations as in Figs 1, 2, 3. White arrows indicate vascular leakage.

We tested the *in-vivo* effects of DPP4-inhibitor on retinal vascular permeability, using a retinopathy of prematurity mouse model. As shown in the experimental scheme (Fig. 7a), neonatal mouse pups were exposed to high oxygen (75%) from day 7 to 12 (situation of hyperoxia) and then were returned to normal air (20%) for 5 days (situation of relative hypoxia). Two different dyes were systemically administered before the harvesting of eyes; TRITC-conjugated lectin from *Bandeiraea simplicifolia* (BS-1 lectin) for vascularity examination, and FITC-dextran for vascular leakage examination. The retinas of postnatal day 17 mice under relative hypoxia showed increased neovascularization, compared to neonatal or postnatal day 12 retina under hyperoxia (Supplementary Fig. S5). Systemic administration of DPP4-inhibitor (DipA) increased not only vascularity (Supplementary Fig. S6) but also vascular leakage (Fig. 7b–d), which was prevented by CXCR4-blocker (AMD3100).

### Retinal capillary leakage due to DPP4-inhibitor: diabetic retinopathy model

**Figure 8:**
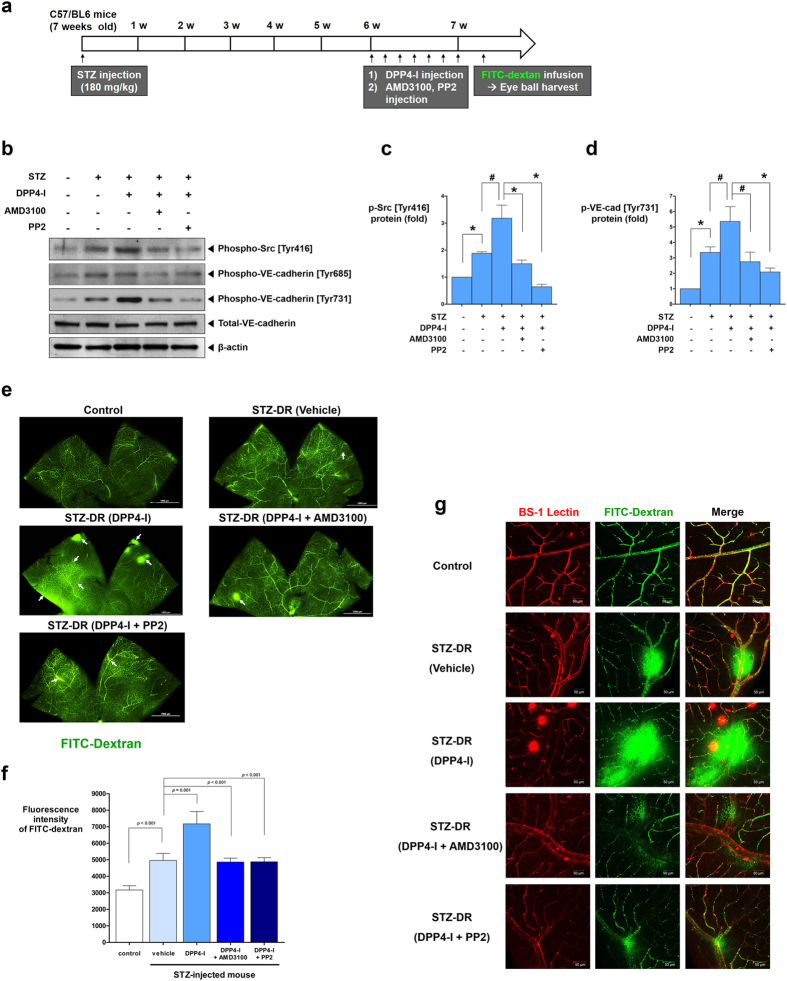
The effects of DPP4-inhibitor on retinal vasculature - diabetic retinopathy model. **(a)** Schematic illustration of the experiment. The experiment consisted of control group (n = 10), STZ-induced (180 mg/kg single dose) diabetic group (n = 9), STZ + DPP4-inhibitor (DipA; 70 μg/kg twice daily) group (n = 12), STZ + DPP4-inhibitor + CXCR4-blocker (AMD3100; 7.5 mg/kg) group (n = 5), and STZ + DPP4-inhibitor + Src-inhibitor (PP2; 1 mg/kg) group (n = 5). **(b)** The phosphorylation of Src [Tyr416] and VE-cadherin [Tyr731] was increased in the retinal tissue of STZ-induced diabetic mouse compared to the control. DPP4-inhibitor further increased the phosphorylation of Src [Tyr416] and VE-cadherin [Tyr731] in the retinal tissue. CXCR4-blocker or Src-inhibitor neutralized the effects of DPP4-inhibitor. **(c,d)** Quantification graphs of the western blot (*p < 0.01, ^#^*p* < 0.05; n = 3 for each group). **(e,f)** Confocal images of the retinal flat mounts and its quantification graph (n = 4–7 for each group). Increased vascular leakage in the retina was observed in the diabetic mice group compared to the control. DPP4-inhibitor further aggravated vascular leakage in the retina which was neutralized by CXCR4-blocker or Src-inhibitor. **(g)** Merged confocal images of the retinal flat mounts. Note the increased vascular leakage after DPP-4 inhibitor treatment. All data are shown as means ± SD. *p* values are determined by Student’s *t* test. STZ: streptozotocin; STZ-DR: streptozotocin induced diabetic retinopathy. Other abbreviations as in Figs 1, 2, 3. White arrows indicate vascular leakage.

A streptozotocin (STZ)-induced diabetic retinopathy model was used to elucidate the *in-vivo* effects of DPP4-inhibitor in actual diabetic mice (Fig. 8a). STZ-injected mice showed significantly higher blood glucose and hemoglobin A1c (HbA1c) levels than did the control group. Body weight was significantly lower in the STZ-injected group (Supplementary Fig. S7). Western blot analysis using the retinal tissue indicated that the phosphorylation of Src [Tyr416] and VE-cadherin [Tyr731] was increased in the STZ-induced diabetic retina, which was further increased by DPP4-inhibitor (DipA) treatment and was decreased by CXCR4-blocker (AMD3100) or Src-inhibitor (PP2) treatment (Fig. 8b–d). VE-cadherin phosphorylation at [Tyr685] was slightly increased by STZ treatment but was not affected by DPP4-inhibitor, CXCR4-blocker, or Src-inhibitor treatment. On retinal vascularity examination, the retinas of STZ-induced diabetic mice showed more vascular leakage than did the control group. DPP4-inhibitor further aggravated retinal vascular leakage in the diabetic retinopathy model. However, CXCR4-blocker and Src-inhibitor treatments reversed the increased vascular leakage induced by DPP4-inhibitor treatment (Fig. 8e–g; see Supplementary Fig. S8 for more representative images).

## Discussion

Here, we demonstrated that DPP4-inhibitor induces (i) the phosphorylation of Src [Tyr 416] and VE-cadherin [Tyr731] and disruption of endothelial cell-to-cell junctions in hECs, thus leading to (ii) endothelial leakage, as demonstrated in several different *in-vitro* and *in-vivo* models. The clinical implications of this study suggested one potential dark side of DPP4-inhibitors, i.e., aggravation of diabetic retinopathy by increasing vascular permeability in the retina. These are, to our knowledge, the first data reporting the relationship between DPP4-inhibitors and increased vascular permeability in retina. Our results are summarized in Supplementary Fig. S9.

### Molecular mechanisms regulating vascular permeability

VE-cadherin is found specifically in endothelial cell adherens junction and is known to play a critical role in controlling angiogenesis and transport across endothelial cell barriers^8^,^9^,^10^,^11^,^12^,^13^,^14^,^31^,^33^,^34^,^35^,^36^,^37^. By inducing H/R conditions, we found that increased SDF-1α mRNA and protein levels led to increased phosphorylation of Src [Tyr416] and VE-cadherin [Tyr731]. We also observed the co-localization of SDF-1α and disruption of VE-cadherin cell-to-cell junctions by immuno-fluorescence analysis. These results suggested that the SDF-1α/CXCR4/Src signaling pathway is involved in VE-cadherin [Tyr731] phosphorylation and the consequent disruption of endothelial cell-to-cell junctions.

DPP4-inhibitors prevent SDF-1α degradation and increase active SDF-1α concentration^20^,^23^,^26^,^27^,^28^,^29^. In our experiments, we demonstrated that VE-cadherin [Tyr731] phosphorylation and disruption of VE-cadherin cell-to-cell junctions were increased by SDF-1α or DPP4-inhibitor treatment and were prevented by CXCR4-blocker or Src-inhibitor treatment. By performing *in-vitro* transwell endothelial permeability and Miles assays, we also found that VE-cadherin cell-to-cell junction disruption, induced by SDF-1α or DPP4-inhibitor, actually led to increased endothelial leakage. In actual *in-vivo* vascular systems, numerous smooth muscle cells surround and interact with endothelial cells^38^. We found an important new example of a paracrine network between hSMCs (main source of SDF-1α) and hECs (main expresser of its receptor, CXCR4) by co-culture transwell assay, as well as an autocrine pathway in hECs monolayer culture experiments.

### Phosphorylation residues of VE-cadherin

Two phosphorylation sites of VE-cadherin [Tyr731 and Tyr685] have been reported to be involved in controlling endothelial permeability^30^,^31^,^35^,^36^,^37^,^39^. Potter *et al.* have reported that cells expressing phosphomimetic mutation at the Tyr731 residue of VE-cadherin show significantly increased endothelial permeability, compared with cells expressing wild-type VE-cadherin^31^. Furthermore, endothelial permeability is not increased in response to vascular endothelial growth factor (VEGF) treatment in cells with phosphorylation-suppressing mutations at the Tyr731 and Tyr658 residues of VE-cadherin^30^. Tyr685 has also been reported to be involved in controlling vascular permeability in a mutational study performed by Wessel *et al.*^36^.

In our western blot analysis, only VE-cadherin [Tyr731] was phosphorylated in response to H/R, SDF-1α, or DPP4-inhibitor treatments, and was reversed by CXCR4-blocker or Src-inhibitor treatments. In contrast, the phosphorylation of [Tyr685] was mildly induced only in the retinas of STZ-induced diabetic mice (Fig. 8b), but it was not affected by H/R, SDF-1α, DPP4-inhibitor, CXCR4-blocker, or Src-inhibitor treatments. Adam *et al.* have reported that phosphorylation of the specific tyrosine residues of VE-cadherin vary depending on the methods used to activate Src^40^. In their experiments, knockdown of C-terminal Src kinase (which suppresses the activity of the Src family kinases) or transduction of constitutively active Src induced the phosphorylation of VE-cadherin [Tyr731] but not of VE-cadherin [Tyr685]. In contrast, VE-cadherin [Tyr685] was phosphorylated in cells transduced with the dominant-negative C-terminal Src kinase. The aforementioned results suggest that Src activation in the absence of C-terminal Src kinase is not sufficient to induce the phosphorylation of VE-cadherin [Tyr685], and VE-cadherin [Tyr685] phosphorylation requires additional events other than Src activation^40^. Furthermore, Baumeister *et al.* have reported that C-terminal Src kinase binds to phosphorylated VE-cadherin [Tyr685] via its SH2 domain and protects the phosphorylated site [Tyr685]^34^. It is possible that the phosphorylation of VE-cadherin [Tyr685] requires an additional signaling cascade (especially C-terminal Src kinase to prevent dephosphorylation of [Tyr685]) other than the SDF-1α/CXCR4/Src signaling pathway activated by DPP4-inhibitor in our experiments.

### Effects of DPP4-inhibitor in murine retinopathy models

It is difficult to observe and evaluate neovascularization in chemically induced diabetic retinopathy models^41^. The retinas of STZ-induced diabetic mice in this study also showed little evidence of retinal neovascularization. In contrast, oxygen-induced retinopathy of prematurity models have demonstrated a reproducible retinal neovascularization suitable for examining neovascularization in the retina^42^. The retinopathy of prematurity model in this study also showed significant neovascularization in the retina. Since retinal neovascularization is the hallmark of proliferative diabetic retinopathy in humans, we used this retinopathy of prematurity model, in addition to a STZ-induced diabetic retinopathy model, to fully evaluate the effects of DPP4-inhibitor on diabetic retinopathy. Our results suggested that DPP4-inhibitor aggravates vascular leakage in the retinas of both models: retinopathy of prematurity and diabetic retinopathy. Centripetal vessel growth in the retinas of postnatal day 17 mice treated with DPP4-inhibitor (Supplementary Fig. S6) suggested that DPP4-inhibitors might also aggravate neovascularization in the retina.

Currently, there is no definite evidence that DPP4-inhibitors cause macular edema or neovascularization in the human retina. However, there have been several studies and case reports describing the association between DPP4-inhibitors (or DPP4 deficiency) and non-cardiac pulmonary edema^43^, pitting edema^44^, angioedema^45^, and peritracheal edema^46^. Whether DPP4-inhibitors provoke symptoms related to increased vascular permeability in human diabetic patients requires further investigation.

### DPP4 inhibition with DipA and gliptins

In randomized trials in humans^32^,^47^, sitagliptin 100 mg, which is the maximal daily dose in clinics, inhibited DPP4 activity by more than 95% at 12 hours after treatment. Doses of 50 mg and 25 mg of sitagliptin resulted in 80–85% and 70–80% inhibition of DPP4 activity, respectively. The weighted average inhibition (area under the DPP4 inhibition-time curve divided by 24 hours) of DPP4 activity was 66.2%, 77.1%, and 88.6% for sitagliptin 25 mg, 50 mg, and 100 mg, respectively^32^. A clinical dose of alogliptin (25 mg) also resulted in 80–85% DPP4 inhibition, which is similar to sitagliptin^48^,^49^. Schwaiger *et al.* have reported that DPP4 activity is inhibited by 48.3%, 52.9%, and 83.9% (mean 61.7%) in three DipA (5 μmol twice daily)-treated mice^50^. A similar degree of DPP4 inhibition was achieved in the myocardial tissue of DipA-treated rats or mice (70 μg/kg twice daily)^20^,^51^. *In-vitro* DipA (5 mM) treatment also resulted in a comparable degree of DPP4 inhibition^52^. Collectively, DipA doses used in animal and *in-vitro* experiments, including our experimental doses, resulted in a similar percentile of DPP4 inhibition as those achieved with the actual clinical doses of gliptins in humans. Furthermore, increased endothelial permeability was observed in our *in-vitro* experiments using 0.1 and 1 μM of sitagliptin (concentrations reflecting the plasma concentrations of patients taking clinical doses of sitagliptin)^32^.

### Diabetic retinopathy and vascular permeability

In addition to conventional treatment, such as laser photocoagulation, anti-angiogenic agents have emerged as a promising treatment option for diabetic retinopathy^53^ because the over-expression of VEGF plays a major role in numerous diseases, including diabetic retinopathy^54^. Intravitreal injection of the anti-VEGF agent bevacizumab reduces the vascular permeability of retinal vessels^55^. The proven efficacy of bevacizumab in diabetic retinopathy indicates that increased vascular permeability plays a significant role in the pathogenesis of diabetic retinopathy. Thus, further clinical research is required to investigate whether the observed vascular leakage after DPP4-inhibition in our study would actually lead to the aggravation of diabetic retinopathy in patients with diabetes.

As an alternative to DPP4-inhibitor, glucagon-like peptide-1 (GLP-1) analog might be considered because it does not inhibit DPP4 or increase SDF-1α levels. GLP-1 receptor agonists, which have an extended half-life, are used for diabetic patients to provide supra-physiological levels of GLP-1 activity. Therefore, on the basis of our results of increased vascular permeability caused by DPP4-inhibitor, GLP-1 agonists might have an advantage over DPP4-inhibitors in terms of concern about aggravation of diabetic retinopathy.

### Limitations

There were several limitations to this study. First, our *in-vivo* experiments were performed using murine, not human, models. *In-vitro* experiments, however, were performed using human cells (hECs and hSMCs). Second, whether the observed *in-vitro* and *in-vivo* effects of DipA and sitagliptin used in this study would actually occur in diabetic patients requires further investigation. Third, it is possible that signaling pathways other than the SDF-1α/CXCR4/Src/VE-cadherin signaling pathway are involved in regulating vascular permeability^56^,^57^.

## Conclusions

In conclusion, our study raised the hypothesis that DPP4-inhibitors might aggravate diabetic retinopathy by increasing vascular permeability through the SDF-1α/CXCR4 axis, followed by Src activation and phosphorylation of VE-cadherin.

## Materials and Methods

### Study design

The objective of our study was to determine the effects of DPP4-inhibitors on diabetic retinopathy. We also investigated the roles of DPP4-inhibitors in regulating vascular permeability. Currently, the role of the SDF-1α/CXCR4/Src/VE-cadherin signaling pathway in regulating vascular permeability has not been clearly established. Therefore, we first explored the role of the SDF-1α/CXCR4/Src/VE-cadherin signaling pathway in regulating vascular permeability by inducing H/R conditions because H/R is a well-established method to increase vascular permeability by inducing loss of endothelial barrier function^58^,^59^. Subsequent *in-vitro* and *in-vivo* experiments were performed to elucidate the effects of DPP4-inhibitors on SDF-1α, CXCR4, Src, VE-cadherin, and vascular permeability. We used the minimum number of animals required to perform sufficient statistical analysis (n = 5–10 for each group). Prespecified endpoints were selected on the pathophysiologic basis of diabetic retinopathy, which involves increased vascular permeability and neovascularization. All of the animal experiments were performed with the approval of the Institutional Animal Care and Use Committee of Seoul National University Hospital. Principles of laboratory animal care (NIH publication no. 85–23, revised 1985; http://grants1.nih.gov/grants/olaw/references/phspol.htm) were followed, as well as specific national laws when applicable. The institutional review board of Seoul National University Hospital approved the isolation of hSMCs from the arteries of the resected stomachs of cancer patients (IRB number: H-1404-007-569). Informed consent was obtained from all of the subjects, and the protocols of this study were consistent with the ethical guidelines of the 1975 Helsinki Declaration.

### Cells and culture conditions of H/R

Human umbilical vein endothelial cells (Lonza, Basel, Switzerland) were plated on 1.5% gelatin (Sigma-Aldrich, St. Louis, MO, USA)-coated dishes and were cultured in EGM-2MV (Lonza, Basel, Switzerland) supplemented with 5% fetal bovine serum. Fourth to sixth passage cells were used in this study. All cells were cultured in 5% CO_2_ at 37 °C. hSMCs were obtained from the mesenteric and gastroepiploic arteries, as described elsewhere^60^. For the H/R experiments, hECs and hSMCs were cultured in hypoxic conditions for 24 hours, followed by 2 hours of reoxygenation conditions. The culture time for hypoxia and reoxygenation was decided on the basis of our previous experiences^58^. For hypoxic conditions, cells were incubated in a hypoxia chamber (Forma Scientific, Midland, ON, Canada), which maintained a low oxygen tension (5% CO_2_ with 1% O_2_, balanced with N_2_).

### Reverse transcriptase-quantitative polymerase chain reaction (RT-qPCR)

Total RNA was prepared using a QIAshredder and an RNeasy mini kit (Qiagen, Hilden, Germany). One microgram of RNA was converted into cDNA according to the PrimeScript™ 1st strand cDNA Synthesis Kit (Takara, Kusatsu, Shiga, Japan). PCR was performed using the SYBR Green PCR Master Mix (Roche, Basel, Switzerland) with specific primers (Supplementary Table S1). Data are presented as relative quantification values. Statistical analysis was performed using normalized cycles to threshold values.

### Western blotting and enzyme-linked immunosorbent assay (ELISA)

Cells were harvested and lysed for 20 minutes in lysis buffer containing protease inhibitors (Roche, Basel, Switzerland). Total protein (10–30 μg) was immunoblotted with primary antibodies against DPP4 (Sigma-Aldrich, St. Louis, MO, USA; goat polyclonal; molecular weight = 110 kDa), CXCR4 (Abcam, Cambridge, UK; rabbit polyclonal; molecular weight = 43 kDa), phospho-Src [Tyr416] (Cell Signaling, Danvers, MA, USA; D49G4; molecular weight = 60 kDa), phospho-VE-cadherin [Tyr731] (Invitrogen, Carlsbad, CA, USA; rabbit polyclonal; molecular weight = 130 kDa), phospho-VE-cadherin [Tyr685] (Abcam, Cambridge, UK; rabbit polyclonal; molecular weight = 140 kDa), total-VE-cadherin (Santa Cruz Biotechnology, Dallas, TX, USA; C-19; molecular weight = 130 kDa), total-Src (Cell Signaling, Danvers, MA, USA; 36D10; molecular weight = 60 kDa), α-tubulin (Santa Cruz Biotechnology, Dallas, TX, USA; YOL1/34; molecular weight = 55 kDa), and β-actin (Santa Cruz Biotechnology, Dallas, TX, USA; C-11; molecular weight = 43 kDa). Two phosphorylation residues were evaluated for VE-cadherin because both [Tyr731]^31^,^35^,^37^,^39^ and [Tyr685]^36^ are known to regulate endothelial permeability. Horseradish peroxidase-conjugated anti-mouse IgM (for α-tubulin; Santa Cruz Biotechnology, Dallas, TX, USA), anti-rabbit IgG (for CXCR4, phospho-Src, and phospho-VE-cadherin; Santa Cruz Biotechnology, Dallas, TX, USA), and anti-goat IgG (for DPP4 and total-VE-cadherin; Santa Cruz Biotechnology, Dallas, TX, USA) antibodies were used as secondary antibodies. Amersham ECL western blotting detection reagents (GE Healthcare Life Sciences, Chicago, IL, USA) were used for detection. Quantification of band intensity was analyzed using TINA software, version 2.0 (RayTest, Straubenhardt, Germany), and was normalized to the intensity of the internal control. ELISA was performed with a Quantikine kit (R&D Systems, Minneapolis, MN, USA).

### Fluorescence-activated cell sorter (FACS) analysis

For flow cytometric analyses, hECs and hSMCs were harvested and fixed with 4% paraformaldehyde. The cells were incubated with anti-DPP4 (Santa Cruz Biotechnology, Dallas, TX, USA) or anti-CXCR4 (Abcam, Cambridge, UK) for 30 minutes on ice and were bound to secondary anti-rabbit Alexa 488 (Invitrogen, Carlsbad, CA, USA). Flow cytometric analysis was performed using BD CantoII (BD Biosciences, Franklin Lakes, NJ, USA).

### Co-culture analysis of hECs and hSMCs

To evaluate the paracrine network between hECs and hSMCs, co-culture experiments of hECs and hSMCs were performed. hECs were cultured in the lower chamber and hSMCs in the upper chamber. hECs were harvested, and western blotting was performed.

### Immuno-fluorescence staining

Cells were fixed with 1% paraformaldehyde for 10 minutes at room temperature. After being washed with PBS and blocked with PBS containing 0.05% Triton-X100 and 1% BSA, the cells were incubated with primary antibodies against anti-VE-cadherin (Santa Cruz Biotechnology, Dallas, TX, USA) and anti-SDF-1α (Abcam, Cambridge, UK). After overnight incubation at 4 °C, the cells were washed with PBS and incubated with Alexa Fluor 555-conjugated donkey anti-goat IgG secondary antibody (for VE-cadherin; Invitrogen, Carlsbad, CA, USA) and Alexa Fluor 488-conjugated donkey anti-rabbit IgG secondary antibody (for SDF-1α; Invitrogen, Carlsbad, CA, USA) for 1 hour at room temperature. The nuclei were counterstained with 4′,6-diamidino-2-phenylindole (DAPI). Fluorescence images were captured with an LSM 710 fluorescence microscope (Zeiss, Oberkochen, Germany). Quantification of fluorescence intensity was performed with MetaMorph (Molecular Devices, Sunnyvale, CA, USA) software or NIS-Elements and ROI statistics software (Nikon, Minato, Tokyo, Japan). Arbitrary units were used.

### *In-vitro* permeability assay

FITC-dextran (40 kDa; Sigma-Aldrich, St. Louis, MO, USA) is an easily detectable tracer. The permeability of the endothelial membrane was assessed by the passage of FITC-dextran through the hECs monolayer. Two days before the experiment, hECs were seeded onto fibronectin-coated 0.4 μm pore 24-well size cell culture inserts (BD Falcon; BD Biosciences, Franklin Lakes, NJ, USA). The cells were cultured in EBM (Lonza, Basel, Switzerland) supplemented with 0.5% fetal bovine serum for starvation under standard culture conditions (37 °C, 95% humidified air and 5% CO_2_) for 18 hours. At the start of the experiment, the culture medium was pre-treated with CXCR4-blocker (AMD3100; Sigma-Aldrich, St. Louis, MO, USA; 1 μg/ml) or Src-inhibitor (PP2; Sigma-Aldrich, St. Louis, MO, USA; 1 μM). DPP4-inhibitor (DipA, Ile-Pro-Ile; Sigma-Aldrich, St. Louis, MO, USA; 100 μM) was applied 30 minutes after CXCR4-blocker or Src-inhibitor treatment. FITC-dextran at a concentration of 20 μg/ml was added to the upper chamber 60 minutes after DPP4-inhibitor treatment (drug treatment time of DipA: 60 minutes). After incubation at 37 °C for 20 minutes, 100 μl of the medium were drawn from the lower chamber (time for permeance: 20 minutes). In order to explore dose-response relationship, multiple doses of DipA (1, 10, 100 μM) and sitagliptin (0.1, 1, 10 μM; MSD, Kenilworth, NJ, USA) was added to the upper chamber. After 30 minutes, FITC-dextran was added to the upper chamber (drug treatment time: 30 minutes). Lower chamber medium was drawn 5 minutes after FITC-dextran treatment (time for permeance: 5 minutes). The fluorescence of the lower chamber was determined by a fluorescence spectro-fluorometer (Tecan Spectra Fluor, LabWrench, Midland, ON, Canada).

### *In-vivo* permeability assay

Miles assay was performed in wild-type C57/BL6 mice. Three groups of mice received an intra-peritoneal injection of DPP4-inhibitor (DipA; 70 μg/kg twice daily), and another group received vehicle for 5 days. The specific dose of DPP4-inhibitor used in this study has been shown to mediate therapeutic effects in murine models^20^,^51^. After 5 days, 2 groups of mice injected with DPP4-inhibitor received an intra-peritoneal injection of CXCR4-blocker (AMD3100; 7.5 mg/kg, once per day) or Src-inhibitor (PP2; 1 mg/kg, once per day). Thirty minutes later, each mouse was injected with PBS into its right ear and SDF-1α (250 ng) to its left ear, and this was followed by an injection of intra-cardiac 0.5% Evans blue dye. Photographs of the ears were obtained, and the mice were euthanized 30 minutes later. Mouse ear tissue was collected with an 8 mm skin punch and was incubated in 300 μl of formamide at 56 °C for 48 hours. The quantity of Evans blue dye in the tissues and standards was determined by assessing the optical density at 600 nm.

### Retinopathy of prematurity model

The oxygen exposure protocol placed oxygen-exposed mouse pups with their nursing mothers in the same covered plastic box with 75% oxygen from postnatal day 7 through postnatal day 12 as previously described^42^. The oxygen was delivered at 75 ± 2%, and it was monitored at least three times daily during the oxygen exposure period. Oxygen concentrations were measured with an oxygen monitor (Teledyne Electronic Technologies, Thousand Oaks, CA, USA). On postnatal day 12, the animals were returned to room air and were subsequently sacrificed by a lethal intra-peritoneal injection of chloral hydrate (360 mg/kg) on postnatal day 17. DPP4-inhibitor injection (DipA; 70 μg/kg, twice daily) was administered from postnatal day 12 to 17. The control mice were injected with PBS in the same manner as DPP4-inhibitor. CXCR4-blocker (AMD3100; 7.5 mg/kg, once per day) was also injected in the same manner as that for the DPP4-inhibitor. BS-1 Lectin (Sigma-Aldrich, St. Louis, MO, USA) was infused systemically for vascularity examination and FITC-dextran (70 kDa; Sigma-Aldrich, St. Louis, MO, USA) for permeability examination. Both eyes of each animal were used for examination of the retinal vascular pattern after flat mounting of the retina.

### Streptozotocin-induced diabetic retinopathy model

To induce diabetes mellitus, 180 mg/kg of intra-peritoneal STZ (Sigma-Aldrich, St. Louis, MO, USA) was injected into 7-week-old C57/BL6 mice. Blood sugar levels (BST; Accu-Chek Performa, Roche Diagnostics, Risch-Rotkreuz, Switzerland) from tail vein blood samples and body weight were monitored weekly. Before the mice were sacrificed, 250 μl of whole blood were drained from the heart for hemoglobin A1c (HbA1c) examination. Two weeks after STZ injection, the mice were confirmed to be diabetic if the BST level was greater than 500 mg/dl. These mice were divided into 4 groups: STZ only; STZ + DPP4-inhibitor; STZ + DPP4-inhibitor + CXCR4-blocker; and STZ + DPP4-inhibitor + Src-inhibitor. Intra-peritoneal DPP4-inhibitor (DipA; 70 μg/kg twice daily) was injected for 7 days beginning from 6 weeks after STZ injection. Single doses of intra-peritoneal CXCR4-blocker (AMD3100; 7.5 mg/kg, once per day) and Src-inhibitor (PP2; 1 mg/kg, once per day) were also injected in the same manner as that for the DPP4-inhibitor. TRITC-conjugated BS-1 Lectin (Sigma-Aldrich, St. Louis, MO, USA) was infused for vascularity examination and FITC-dextran (70 kDa; Sigma-Aldrich, St. Louis, MO, USA) for permeability examination. Both eyes of each mouse were used for examination of the retinal vascular pattern after flat mounting of the retina. Images were obtained with a Nikon DS-Qi2 CMOS camera head mounted on a Nikon Ti-E motorized inverted microscope. To quantify the fluorescence intensity of FITC-dextran, captured images from each experiment were analyzed using NIS-Elements and ROI statistics software (Nikon, Minato, Tokyo, Japan). Using the black-green interface, we created region of interest on each captured image. By ROI statistics, we calculated the mean intensity of FITC-dextran in the region of interest of each captured image. After the retinal vascularity examination, western blot analysis for phosphorylated Src and VE-cadherin was performed using the retinal tissue.

### Statistics

Statistical analysis was conducted using SPSS software, version 18.0 (SPSS Inc., Chicago, IL, USA). Values are expressed as means ± standard deviations. Continuous variables were compared with the unpaired t-test or analysis of covariance with post hoc analysis using Bonferroni’s correction. All tests of significance were two-tailed, and *p* values < 0.05 were considered to indicate statistical significance.

## Additional Information

**How to cite this article**: Lee, C.-S. *et al.* Dipeptidyl Peptidase-4 Inhibitor Increases Vascular Leakage in Retina through VE-cadherin Phosphorylation. *Sci. Rep.* **6**, 29393; doi: 10.1038/srep29393 (2016).

## Supplementary Material

Supplementary Information
