# Supplementary material for: Dipeptidyl Peptidase-4 Inhibitor Increases Vascular Leakage in Retina through VE-cadherin Phosphorylation
Source: Sci Rep. 2016 Jul 6;6:29393. doi: 10.1038/srep29393 (PMC4933943; doi:10.1038/srep29393)
Supplement: Supplementary Information [file srep29393-s1.pdf]

Dipeptidyl Peptidase-4 Inhibitor Increases Vascular Leakage in Retina through VE-cadherin Phosphorylation

Choon-Soo Lee, PhD,[1,2,4]* Yun Gi Kim, MD,[3]* Hyun-Jai Cho, MD,[1,2,3]

Jonghanne Park, MD,[1,2,3] Heewon Jeong, BS,[1] Sang-Eun Lee, MD,[1,2,3]

Seung-Pyo Lee, MD,[3] Hyun-Jae Kang, MD,[2,3] Hyo-Soo Kim, MD[1,2,3,4]

[1]National Research Laboratory for Stem Cell Niche, Seoul National University College of Medicine,

[2]Innovative Research Institute for Cell Therapy, Seoul National University Hospital,

[3]Cardiovascular Center & Department of Internal Medicine, Seoul National University Hospital,

[4]Department of Molecular Medicine and Biopharmaceutical Sciences, Graduate School of Convergence Science and Technology, and College of Medicine or College of Pharmacy, Seoul National University, Seoul, Korea

Contact information

Address for correspondence: Hyo-Soo Kim, MD, PhD

Director of National Research Laboratory for Cardiovascular Stem Cell Niche,

Professor, Department of Internal Medicine, Seoul National University Hospital,

101 Daehak-ro, Jongno-gu, Seoul 110-744, Korea

Tel: 82-2-2072-2226 / Fax: 82-2-766-8904

E mail: hyosoo@snu.ac.kr or usahyosoo@gmail.com

*: The first two authors contributed equally to this work.

Figure S1

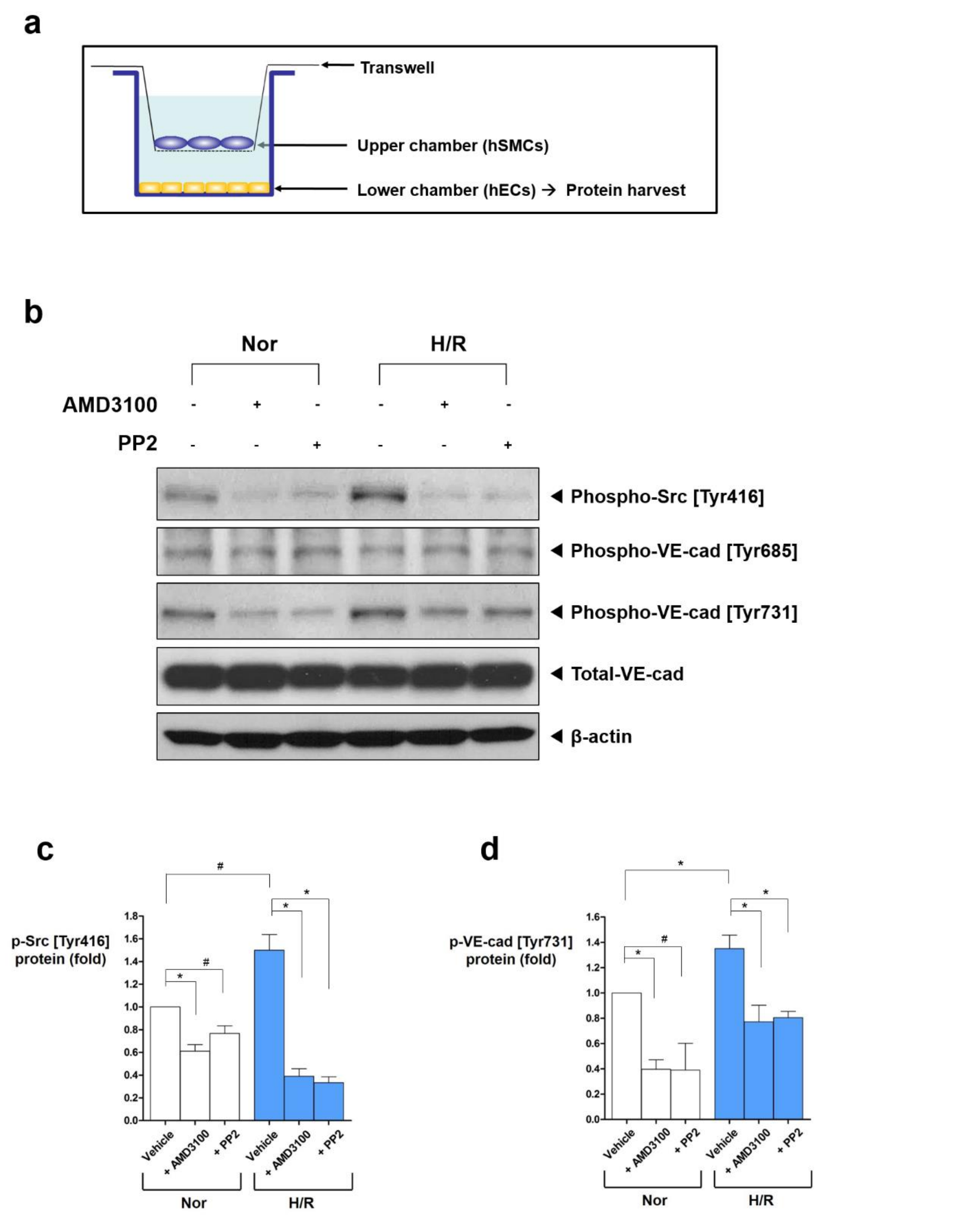

Figure S1. Co-culture experiment of hECs and hSMCs.

In-vitro co-culture experiment was performed to simulate the paracrine network between hSMCs (main source of SDF-1α) and hECs (having its receptor CXCR4). H/R on hSMCs increased the

phosphorylation of Src [Tyr416] and VE-cadherin [Tyr731] in hECs, which was prevented by CXCR4-blocker (AMD3100; 1 μg/ml) or Src-inhibitor (PP2; 1 μM).

(a) Experimental scheme of the co-culture experiment.

(b) H/R increased the phosphorylation of Src [Tyr416] and VE-cadherin [Tyr731] in hECs under the influence of SDF-1α secreted from hSMCs.

(c, d) Quantification graphs of the western blot (*: $p < 0.01$, $^{\#}$: $p < 0.05$; n = 3 for each group).

All data are shown as means ± SD. *p* values are determined by Student's *t* test.

hECs: human endothelial cells; H/R: hypoxia/reoxygenation; hSMCs: human smooth muscle cells; Nor: normoxia; p-Src: phosphorylated Src; p-VE-cad: phosphorylated VE-cadherin; SD: standard deviations; SDF-1α: stromal cell derived factor 1α; VE-cad and VE-cadherin: vascular endothelial-cadherin.

Figure S2

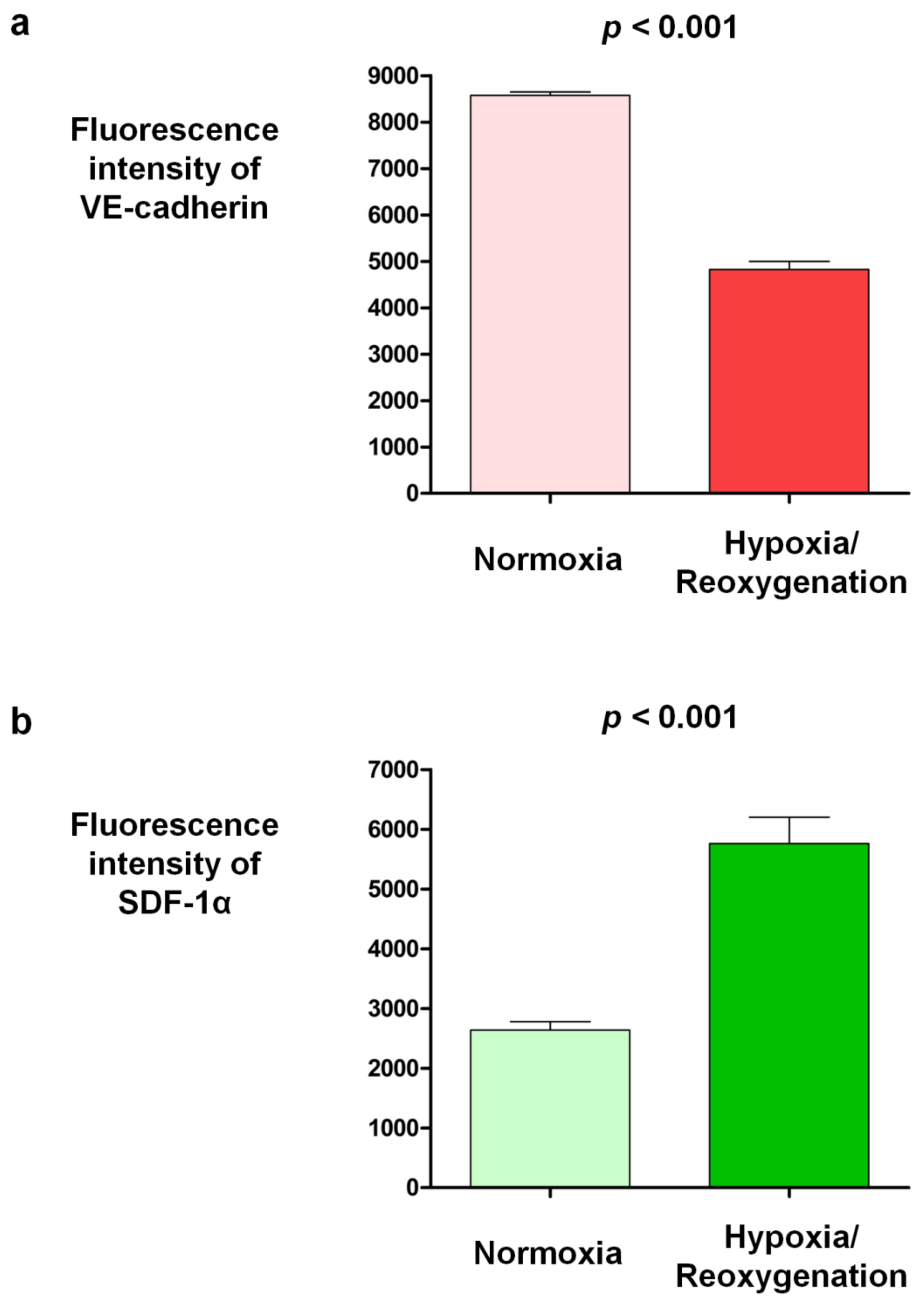

Figure S2. Activation of Src kinase during H/R decreased VE-cadherin expression and increased SDF-1α expression.

Quantification graphs showing fluorescence intensity of VE-cadherin (**a**; n = 6 for each group) and

SDF-1α (**b**; n = 5 for each group) of Fig. 2c. All data are shown as means ± SD. *p* values are determined by Student's *t* test.

H/R: hypoxia/reoxygenation; SD: standard deviations; SDF-1α: stromal cell derived factor 1α; VE-cadherin: vascular endothelial-cadherin.

Figure S3

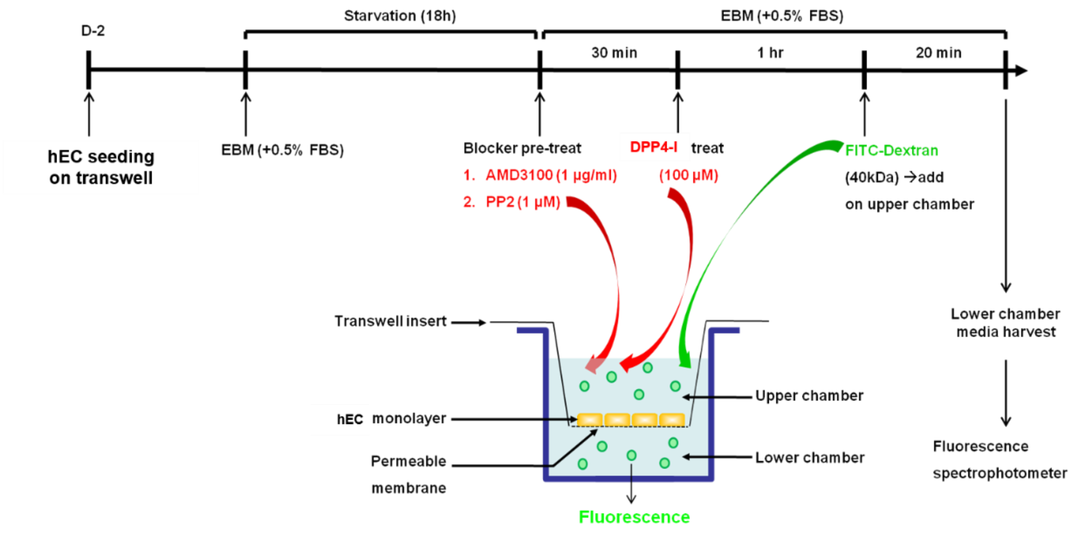

Figure S3. Schematic illustration of the in-vitro transwell endothelial permeability assay.

The fluorescence of the lower chamber was determined by a fluorescence spectro-fluorometer (Tecan Spectra Fluor) according to the manufacturer's protocol.

Figure S4

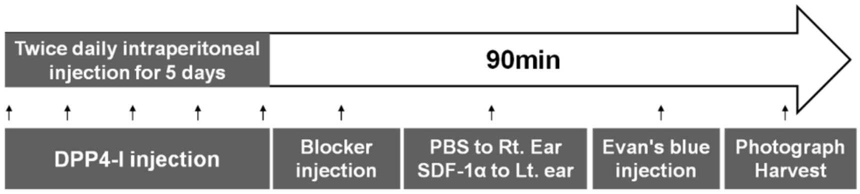

Figure S4. Schematic illustration of the Miles assay.

Four groups of mice had an intra-peritoneal injection with either vehicle, DPP4-inhibitor (DipA; 70 μg/kg twice daily), DPP4-inhibitor + CXCR4-blocker (AMD3100; 7.5 mg/kg), or DPP4-inhibitor + Src-inhibitor (PP2; 1 mg/kg). Each mouse was injected with PBS to the right ear and SDF-1α (250 ng) to the left ear.

DipA: diprotin A; DPP4-I and DPP4-inhibitor: dipeptidyl peptidase 4-inhibitor; SDF-1α: stromal cell derived factor 1α.

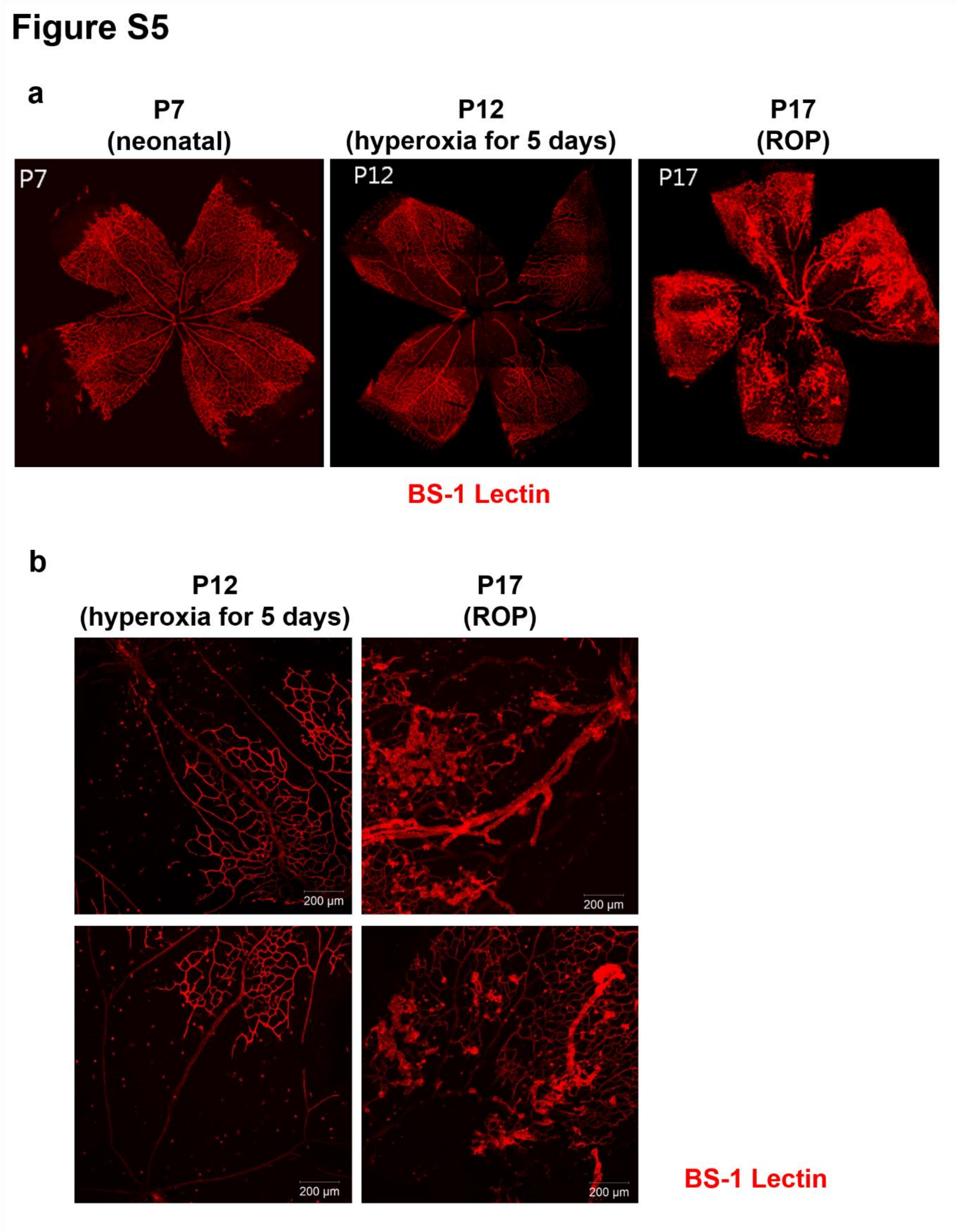

Figure S5. Aberrant vessel growth in ROP model.

(a, b) Aberrant vessel growth was observed in the retinas of postnatal day 17 mice compared to the postnatal day 7 and 12 mice.

BS-1 lectin: *Bandeiraea simplicifolia* lectin 1; P7: postnatal day 7; P12: postnatal day 12; P17: postnatal day 17; ROP: retinopathy of prematurity.

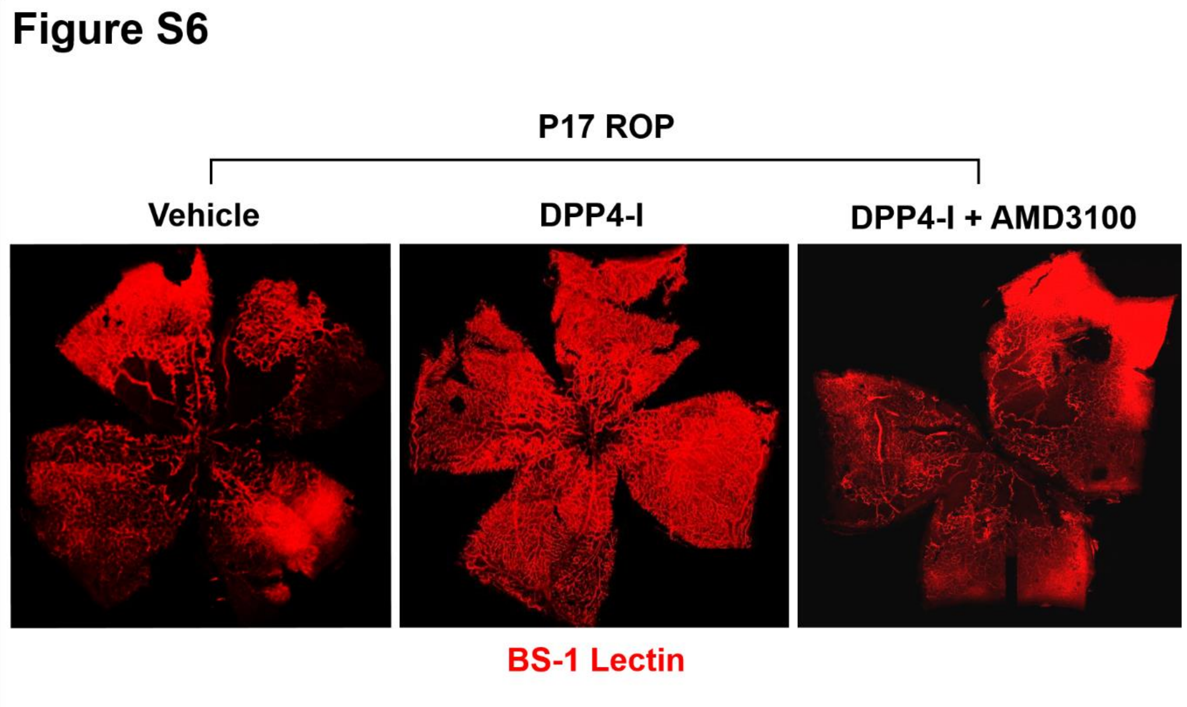

Figure S6. Increased neovascularization after DPP4-inhibitor treatment.

Compared with the vehicle, DPP4-inhibitor (DipA; 70 µg/kg twice daily) enhanced the centripetal vessel growth in the postnatal day 17 retinas, which was prevented by CXCR4-blocker (AMD3100; 7.5 mg/kg).

BS-1 lectin: *Bandeiraea simplicifolia* lectin 1; DipA: diprotin A; DPP4-I and DPP4-inhibitor: dipeptidyl peptidase 4-inhibitor; P17: postnatal day 17; ROP: retinopathy of prematurity.

Figure S7

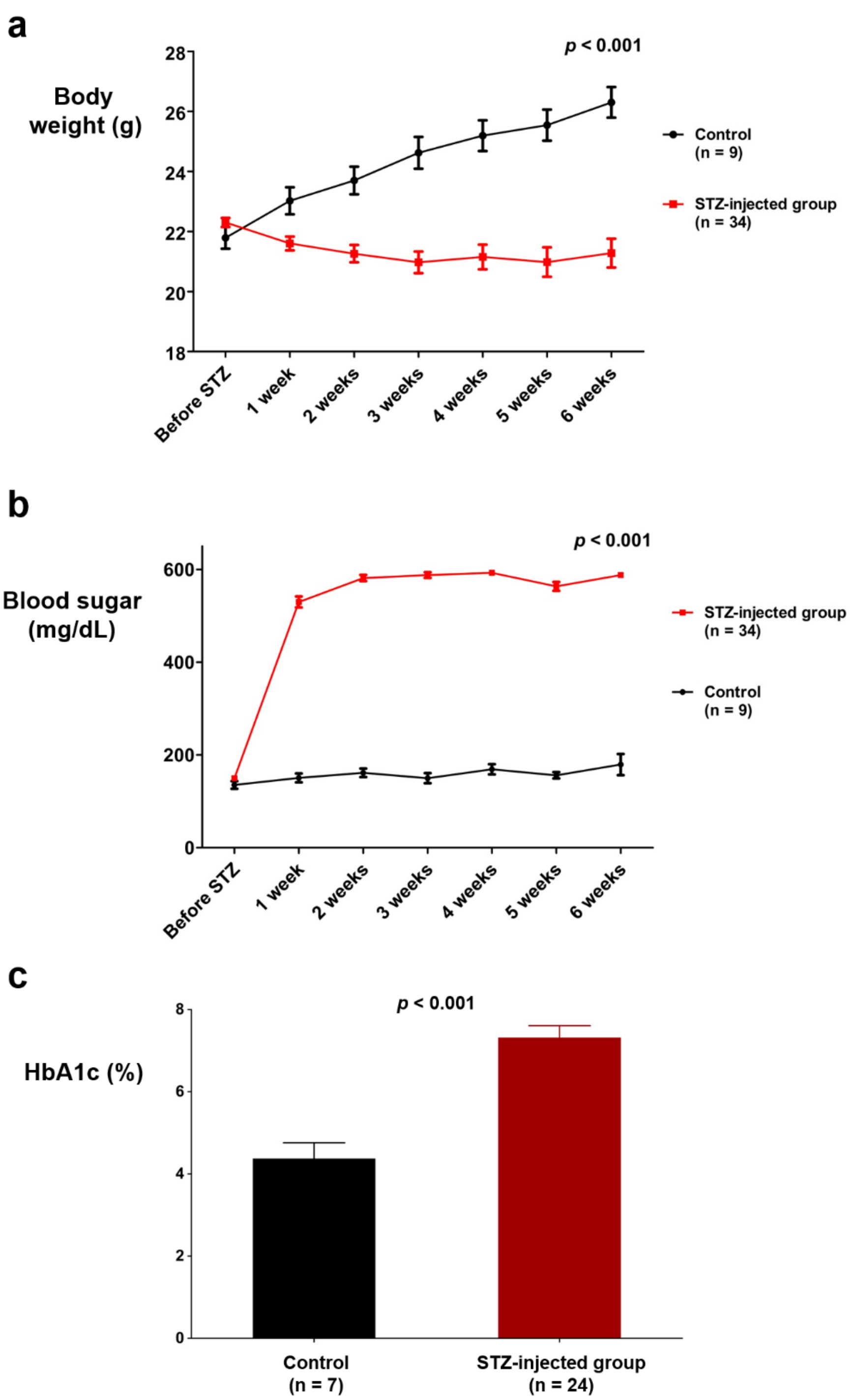

Figure S7. Body weight, blood glucose, and HbA1c changes in STZ-induced diabetic mice.

STZ-induced diabetic mice showed significantly lower body weight (**a**) compared to the control. Blood glucose (**b**) and HbA1c level (**c**) was significantly higher in STZ-induced diabetic mice.

All data are shown as means ± SE. *p* values are determined by Student's *t* test.

HbA1c: hemoglobin A1c; SE: standard errors; STZ: streptozotocin.

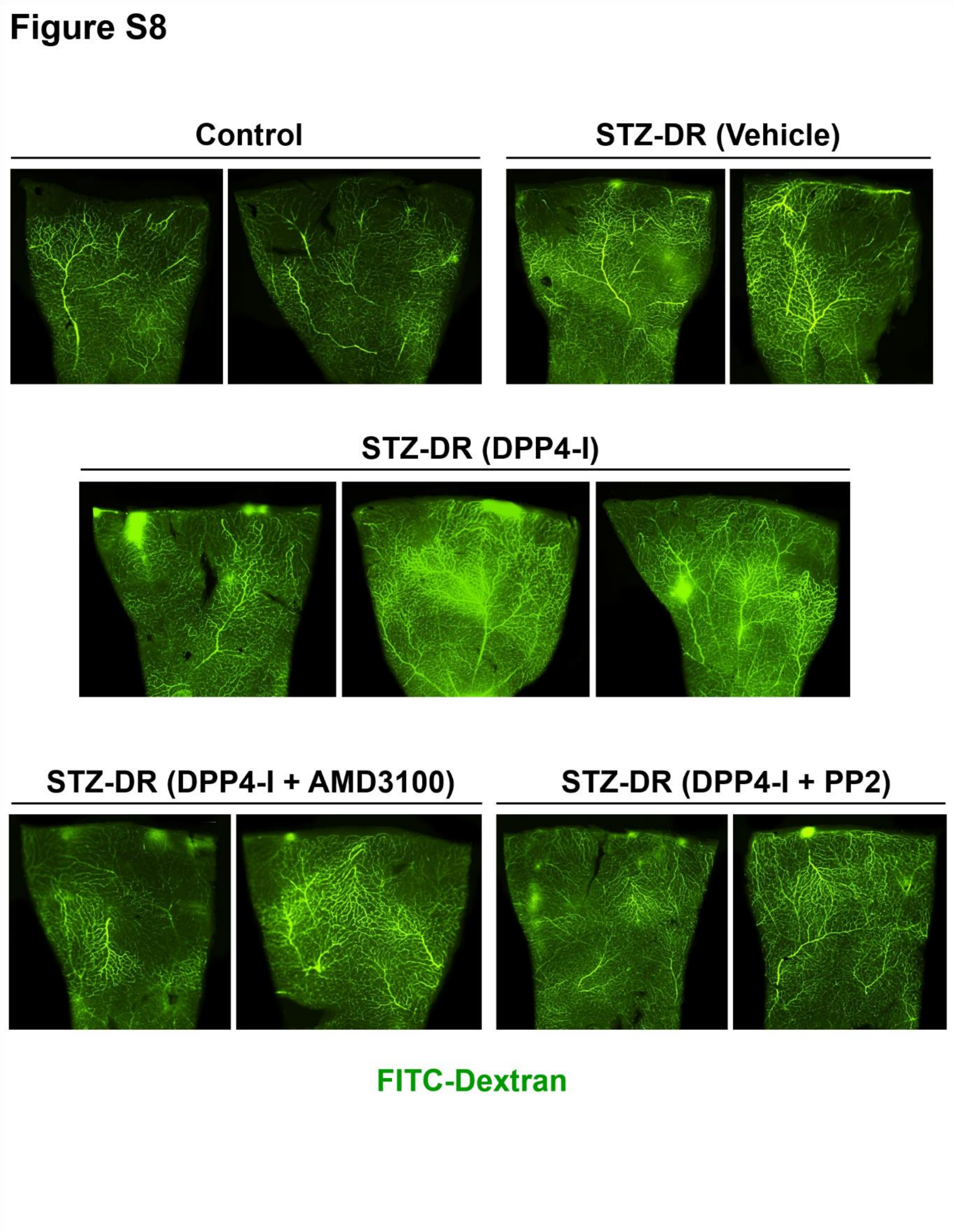

Figure S8. DPP4-inhibitor aggravated vascular leakage in the retinas of diabetic mice. DPP4-inhibitor (DipA; 70 μg/kg twice daily) increased vascular leakage in the retinas of diabetic mice. CXCR4-blocker (AMD3100; 7.5 mg/kg) and Src-inhibitor (PP2; 1mg/kg) neutralized the effects of DPP4-inhibitor.

DPP4-I and DPP4-inhibitor: dipeptidyl peptidase 4-inhibitor; FITC-dextran: fluorescein isothiocyanate conjugated-dextran; STZ: streptozotocin; STZ-DR: streptozotocin induced diabetic retinopathy.

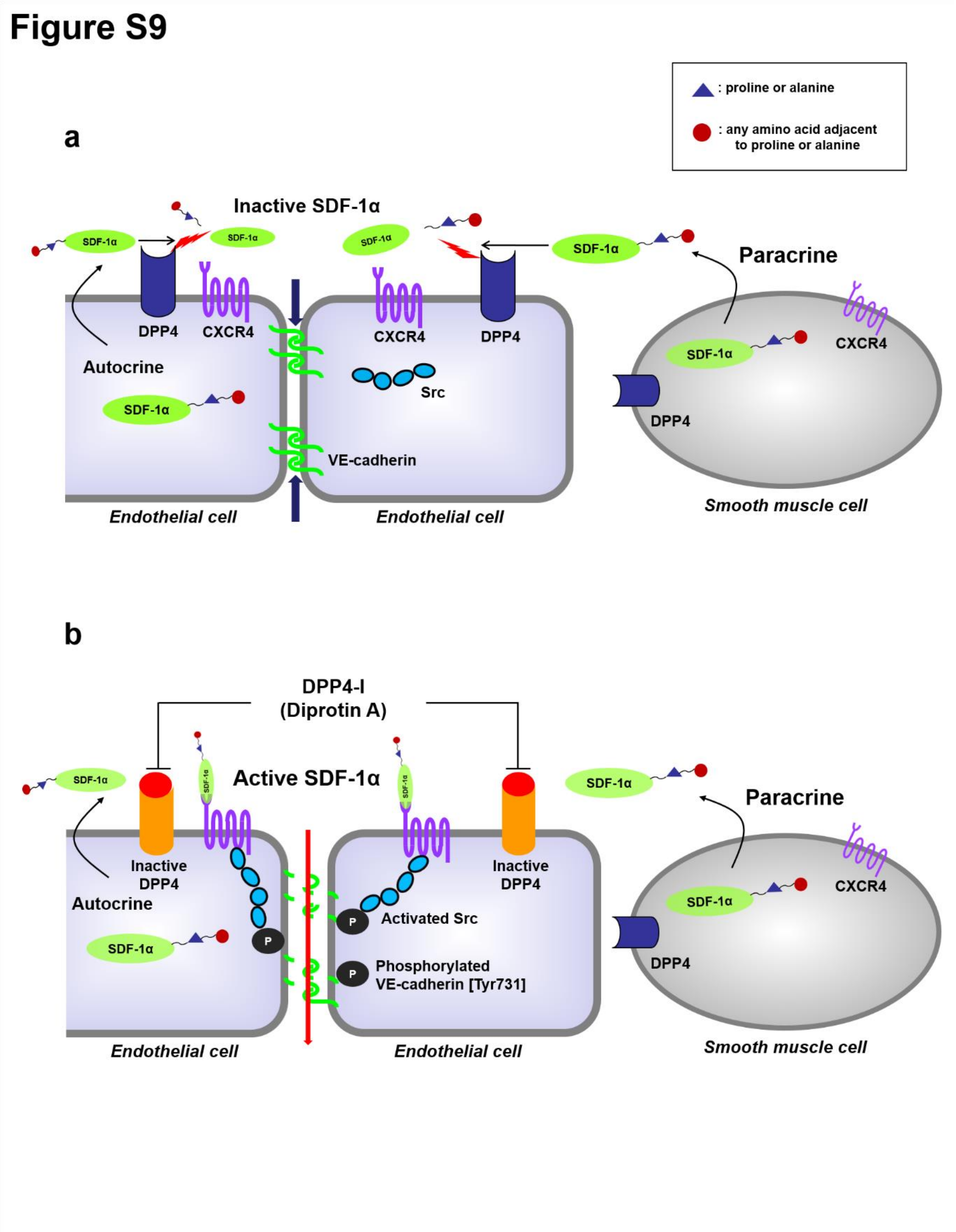

Figure S9. Schemes of how DPP4-inhibitors induce vascular leakage.

(a) In normal state, SDF-1α secreted from vascular smooth muscle cells or endothelial cells is degraded by DPP4, resulting in shut-down of Src and VE-cadherin phosphorylation and maintenance

of cell-to-cell junction integrity.

(b) DPP4-inhibition increases concentration of active SDF-1α through blocking its degradation. As a result, the SDF-1α/CXCR4/Src/VE-cadherin signaling pathway is activated leading to vascular leakage.

DPP4: dipeptidyl peptidase 4; DPP4-I and DPP4-inhibitor: dipeptidyl peptidase 4-inhibitor; SDF-1α: stromal cell derived factor 1α; VE-cadherin: vascular endothelial-cadherin.

Table S1. RT-qPCR primer sequences.

Genes	Sense (S) /Antisense (AS)	Sequences
h-SDF-1α	S	CCAAACTGTGCCCTTCAGAT
	AS	CCACTTTAGCTTCGGGTCAA
h-CXCR4	S	TGACTTTGAAACCCTCAGCG
	AS	CCTCCCCATCTTTTCCCATA
h-DPP4	S	AGAATGTCCAGATGCCCTCC
	AS	TTGACTACATGGGCCTGCAT
h-GAPDH	S	AACATCATCCCTGCCTCTAC
	AS	CCCTGTTGCTGTAGCCAAAT

DPP4: dipeptidyl peptidase 4; RT-qPCR: reverse transcriptase-quantitative polymerase chain reaction; SDF-1α: stromal cell derived factor-1α.
